# Research on Isomorphic Task Transfer Algorithm Based on Knowledge Distillation in Multi-Agent Collaborative Systems

**DOI:** 10.3390/s24144741

**Published:** 2024-07-22

**Authors:** Chunxue Bo, Shuzhi Liu, Yuyue Liu, Zhishuo Guo, Jinghan Wang, Jinghai Xu

**Affiliations:** School of Physics and Electronic Engineering, Qilu Normal University, Jinan 250200, China

**Keywords:** multi-agent collaborative systems, domain separation network, knowledge distillation, isomorphic task transfer

## Abstract

In response to the increasing number of agents and changing task scenarios in multi-agent collaborative systems, existing collaborative strategies struggle to effectively adapt to new task scenarios. To address this challenge, this paper proposes a knowledge distillation method combined with a domain separation network (DSN-KD). This method leverages the well-performing policy network from a source task as the teacher model, utilizes a domain-separated neural network structure to correct the teacher model’s outputs as supervision, and guides the learning of agents in new tasks. The proposed method does not require the pre-design or training of complex state-action mappings, thereby reducing the cost of transfer. Experimental results in scenarios such as UAV surveillance and UAV cooperative target occupation, robot cooperative box pushing, UAV cooperative target strike, and multi-agent cooperative resource recovery in a particle simulation environment demonstrate that the DSN-KD transfer method effectively enhances the learning speed of new task policies and improves the proximity of the policy model to the theoretically optimal policy in practical tasks.

## 1. Introduction

In machine learning, multi-agent collaborative systems abstract real-world objects into agents (such as drones, robots, traffic signals, etc.), each endowed with certain perception, decision-making, and action capabilities [1,2]. These agents then seek optimal solutions to tasks or achieve specific goals by solving for each agent’s optimal execution strategy through certain paradigms [3]. With the continuous advancement of computer technology, research on multi-agent collaborative systems has garnered increasing attention and become a focal point in the field of artificial intelligence. To date, multi-agent collaborative systems have been widely applied in various domains including economics, industry, society, military, and have had profound impacts [4,5].

The prevalent approach to solving multi-agent collaborative problems involves using deep reinforcement learning methods. Reinforcement learning employs a Markov decision framework to model multi-agent collaborative systems, where learning agents jointly optimize a single reward signal [6,7]. Each agent selects actions from feasible strategies based on its local observations and receives rewards accordingly, aiming to maximize the reward signal value derived from executing actions. The introduction of deep neural networks optimizes the process from environmental observation information to action policy execution for intelligent agents. By optimizing their structural parameters based on reward signal information, agents attain optimal policy action outputs. Intuitively, the behavior policies of agents are abstracted as deep neural networks, which take environmental observations as inputs and produce actions, continuously refining the network through trial-and-error interactions with the environment to achieve optimal decision-making [8]. This characteristic endows multi-agent reinforcement learning with adaptability to specific environments, enabling it to solve various practical multi-agent collaborative application problems.

However, multi-agent collaborative systems face increasingly complex task environments, posing numerous challenges. In practical applications, due to the instability of environmental data, intelligent agents typically require sampling a large number of random interaction samples to learn approaches close to optimal strategies in the absence of domain expert demonstrations as prior knowledge, resulting in significant resource consumption and inherent risks. Additionally, whenever task objectives or environmental states change, all learning processes must restart due to fixed policy structures [9,10]. These practical issues prompt us to utilize knowledge from solved problems to improve the learning process of intelligent agents in new tasks. Therefore, researching knowledge transfer in multi-agent collaborative systems holds significant practical significance.

In multi-agent collaborative tasks, the direct correlation between the state space and the number of agents leads to a deep coupling with the Markov Decision Process (MDP), making it impossible to achieve the desired results by directly using previously trained policy models when the number of agents changes. On the one hand, existing agent policy networks are unable to fully adapt to environmental drift to some extent, and on the other hand, new agents need to learn from scratch; both are challenges that reinforcement learning algorithms struggle to overcome [11]. Introducing transfer learning into reinforcement learning scenarios allows agents to utilize knowledge from historical experience during training, thereby expediting the learning process for new tasks. However, due to the complex nature of reinforcement learning algorithms, the transfer process poses numerous challenges.

Unlike the handling of input images in computer vision, the network input for non-image-based reinforcement learning tasks is closely related to the characteristics of the environment. Changes in the number of agents and environmental entities alter the dimensions of the network’s inputs, rendering the trained original model unable to directly transfer to the target network model [12,13]. Fine-tuning, where certain network layers are fixed while others are adjusted and retrained, is a viable approach. However, while determining how to fine-tune corresponding network layers, the differential impact of task variations in input dimensions needs to be considered. Moreover, during the learning process for the target task, the original policy network may be disrupted, losing the characteristics of the source task, resulting in poor transfer effects for a wide range of reinforcement learning tasks [14]. Compared to fine-tuning pre-trained models to achieve model reuse, extracting abstract knowledge from pre-trained agents in the source domain through the powerful performance of neural networks to guide training in the target domain can reduce the exploration space for agents in the target domain, prevent unnecessary blind exploration, and promote training in a direction beneficial to the target task. This approach is a commonly used transfer method. However, the key challenge of this approach lies in how to extract valuable knowledge from the source and target domains to ensure positive guidance for agents during the transfer process without causing negative transfer [15,16].

Addressing the aforementioned issues, this study explores a knowledge transfer framework for multi-agent knowledge transfer based on knowledge distillation, combined with the performance-enhancing domain separation network, and validates it in a multi-agent simulation environment. Experimental results in multiple collaborative scenarios such as drone surveillance, drone cooperative target occupation, drone cooperative material recovery, and robot cooperative box pushing demonstrate that the introduction of domain separation networks effectively corrects the supervisory information generated by pre-trained agents during the knowledge distillation process. In the learning of new tasks, the improved knowledge distillation method proposed in this study (DSN-KD) enhances the learning speed of agents by refining the behavioral information and improves the optimal performance of the policy model.

## 2. Related Works

In scenarios involving isomorphic task transfer, the source and target tasks maintain consistency in state variables and action spaces, while variations can exist in the range of state variable values, environmental dynamics, and reward function settings. Due to the minimal differences between tasks and maintaining isomorphism in overall properties, knowledge can be directly transferred using state features and actions.

Early reinforcement learning transfer methods assumed stricter settings for isomorphic tasks, requiring complete equivalence in state spaces. Transfer methods directly migrated shallow knowledge such as value functions and models from the source task. For instance, Huang et al. [17] altered environmental dynamics in robot control and tracking tasks to design source and target tasks, achieving transfer through value function initialization. Similarly, James et al. [18] changed initial states in vision-based robot behavioral tasks to introduce task differences, followed by value function transfer. Nussenbaum et al. [19] constructed locally weighted regression models for state transition processes to facilitate transfer; however, this method is limited to reinforcement learning methods with known environmental dynamics models. Gavini et al. [20] proposed a method based on maximum-margin transfer. These methods laid the groundwork for understanding how isomorphic tasks could benefit from direct knowledge transfer. However, their reliance on strict isomorphism limited their applicability in more varied and practical scenarios.

In cases where the state space undergoes only changes in value range rather than complete equivalence, which aligns more with general practical applications, transfer methods primarily focus on leveraging source task knowledge suitable for the target task, achieved through additional computation or abstract implementations of transfer [21]. Among these, the transfer of partial policies receives the most attention. Chai et al. [22] explored how effective control knowledge transfer could be achieved through learning skills and hierarchical representation structures. Brito et al. [23] studied how transferring subgoal policies could facilitate reinforcement learning transfer in robot navigation tasks. Bomanson et al. [24] proposed an option framework based on state transitions, termed relative options, by examining how to choose appropriate state transitions for transfer and using a state transition-based algorithm for selecting optimal relative options. Qiu et al. [25] introduced a programmable reinforcement learning agent based on state abstraction, transferring similar state subspaces to the target task. Zhang et al. [26] investigated rule-based experience transfer across environments of varying difficulty levels. Xu et al. [27] transferred rules learned from the source task to aerial combat mission scenarios. Unlike policy transfer, An et al. [28] simplified the action set by removing suboptimal actions from the source task to guide the exploration of agents in the new task. Gao et al. [29], inspired by knowledge distillation methods, used pre-trained agent policy model outputs as teacher knowledge to distill target task agents. These advancements demonstrate how transfer learning can be applied in more flexible and practical ways, moving beyond the constraints of strict isomorphism.

Research on isomorphic task transfer in multi-agent collaborative systems has made some progress, addressing common issues such as:

1. Complexity of transfer process design: The goal of transfer learning is to reduce training costs. However, expert-designed mappings between different task domains can lead to significant workload and cost overheads. This limits the reinforcement learning models to the capabilities of the designers, as seen in AI systems like AlphaGo, which would be nearly impossible to achieve through transfer alone.
2. Highly limited transfer models: Some methods focus solely on transferring between two specific tasks with explicit mappings, resulting in limited and often unidirectional transfer.

To address these challenges, this paper proposes a knowledge distillation method combined with domain separation networks, conducting corresponding experiments to demonstrate its effectiveness. This approach aims to reduce the complexity of the transfer process and expand the applicability of transfer models in multi-agent collaborative systems. The proposed method introduces several novel aspects compared to the state-of-the-art:

1. Integration of knowledge distillation with domain separation networks: This combination allows for more efficient transfer of knowledge between tasks by separating transferable knowledge from task-specific features.
2. Scalability in multi-agent systems: Unlike traditional methods, our approach is designed to scale with the increasing number of agents, maintaining the effectiveness of collaborative strategies.
3. Flexibility in practical applications: The method is not constrained by strict isomorphism, making it suitable for a wider range of practical scenarios where state spaces and action spaces may vary.

By addressing these key issues, our method offers a significant advancement in the field of reinforcement learning transfer, providing a more versatile and efficient approach for knowledge transfer in multi-agent systems.

## 3. The Proposed Method

### 3.1. Overall Method Design

**Figure 1:**
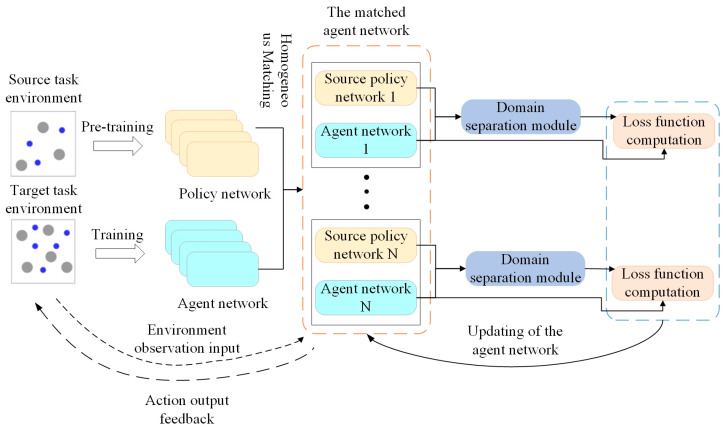
The framework of DSN-KD.

To address the issue of poor adaptability of existing strategies in multi-agent systems with isomorphic fluctuations in agent numbers, this paper proposes the DSN-KD transfer method, which combines domain separation networks with the knowledge distillation method. The model mainly consists of the policy network alignment, domain separation module, and loss calculation components, as shown in Figure 1.

Initially, the structure of the pre-trained policy network in the source task is adjusted and expanded to pair with the intelligent agent network in the target task. Paired agents and policy networks share the same environmental observation input. During the training of intelligent agents in the target environment, the source policy network remains fixed. After receiving observation input in each round, the feature output before the action mapping layer is utilized by both paired policy networks and intelligent agent networks to compute common features of agent policies in similar tasks. This process is completed by the domain separation module, which updates based on the loss functions of its different components. Finally, the transfer loss is calculated based on the common features and the observation output of the target intelligent agent for network updates. The subsequent sections will provide a detailed analysis of the policy allocation, domain separation module, and loss function calculation components in the framework.

### 3.2. Analysis of Transfer Framework Based on Improved Knowledge Distillation

**Figure 2:**
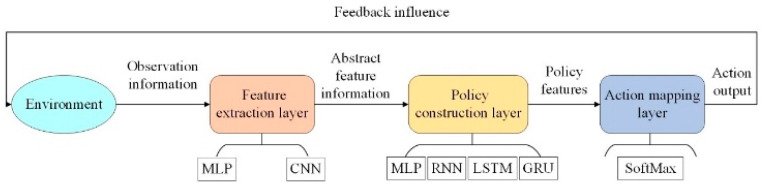
Agent policy network.

In the Actor-Critic framework of reinforcement learning algorithms, the actor network serves as the executor of the agent’s policy. The agent directly executes corresponding actions in the environment through the actor network. The network structure can theoretically be divided into three parts: the feature mapping layer, the intermediate layer, and the action mapping layer. The feature mapping layer receives the environmental state inputs observed by the agent and maps the state input vector to a higher-level abstract space for subsequent analysis and computation by the neural network. The intermediate layer is responsible for processing the abstract features extracted by the feature mapping layer and constructing the policy for the target task through a multi-layer network. The action mapping layer, located at the terminal part of the entire network structure, outputs actions to the environment. Typically, the feature mapping layer is designed using multi-layer perceptrons and convolutional neural networks, while the policy construction layer employs structures such as multi-layer perceptrons, recurrent neural networks (RNNs), long short-term memory neural networks (LSTMs), and gated recurrent units (GRUs). The action mapping layer generally uses softmax to map input features to the execution probabilities of different actions in the action space. An example of a policy network is shown in Figure 2.

**Figure 3:**
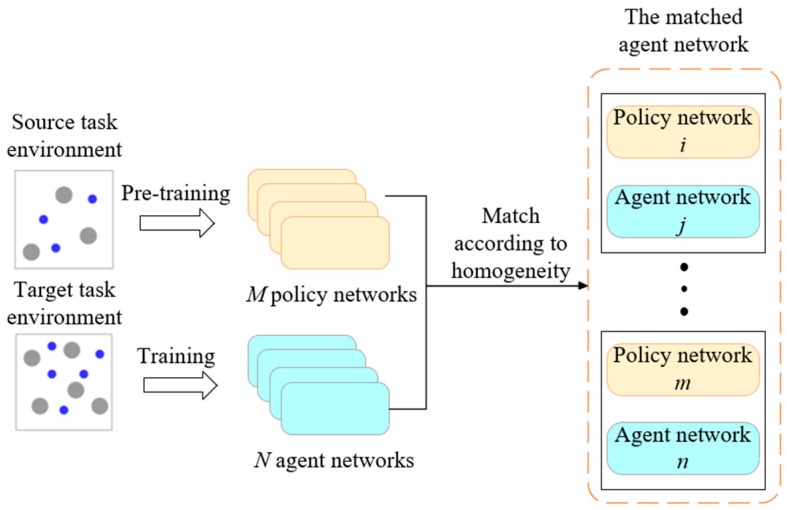
Pairing of pre-trained policy networks with target intelligent agents.

In the initial stage of transfer, it is necessary to pair the pre-trained policy model with the intelligent agents in the target environment. This process essentially involves establishing a pairing between a teacher model and a student model. In the transfer of homologous tasks, the categories and properties of intelligent agents remain unchanged, allowing the policy networks of agents from the source task to be directly matched one-to-one with those of corresponding agents in the target task. This process is illustrated in Figure 3. In the target task, each intelligent agent will be paired with a pre-trained policy network from the source task. The pre-trained policy network will serve as the teacher network to impart prior knowledge from the source task training to the corresponding policy network of the intelligent agent in the target environment. As the number of intelligent agents in the source and target tasks often differs, repeated matching of homologous agent policy networks is necessary to achieve one-to-one knowledge transfer.

During the training process of intelligent agents in the target environment through reinforcement learning algorithms, the observation results of the target intelligent agents are simultaneously input into both the paired pre-trained policy model and the current policy model. Based on identical observation inputs, the input features of the action mapping layers of both are used to construct transfer losses, measuring the gap between tasks. Meanwhile, the current target intelligent agents also receive feedback through interaction with the environment. Through this approach, the intelligent agents in the target task not only maximize the overall rewards from the environment but also emulate the outputs of well-trained policy models from the source task based on similarities between tasks.

**Figure 4:**
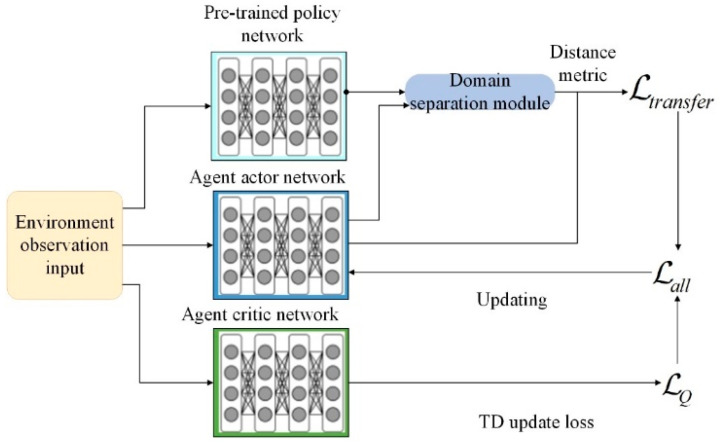
Training process of target intelligent agents.

The transfer loss originates from the distance between the shared behavioral features of tasks and the current policy features of intelligent agents. The extraction of task common features will be elaborated in detail in the next section. A key step in the training process is to combine the differences in policy features between the two and the environmental rewards. Typically, a hyperparameter is used to adjust the relationship between them:

(1) $$L_{all}=\alpha L_{transfer}+\left(1-\alpha \right)L_{Q}$$

where $L_{all}$ represents the final loss used for updating the policy network of the target intelligent agents, $L_{transfer}$ measures the discrepancy between the teacher policy network and the policy network of the target intelligent agents, represents the loss obtained by the target intelligent agents interacting with the environment under the reinforcement learning framework, and α is a weight that balances the two, with a value range of [0, 1]. The overall procedure is illustrated in Figure 4, with the design of the domain separation module to be analyzed in the next section.

In the process of knowledge transfer, the concept of knowledge distillation is employed, where the teacher model used to transmit knowledge is a well-trained model trained on the same dataset as the current student model. To extract knowledge, a certain discrepancy loss is needed to minimize the gap between them. Let $a_{p}$ and $a_{c}$ be the teacher output features and the input features of the final action mapping layer in the target intelligent agent’s policy model, respectively, where $a_{p}=\left[a_{1},a_{2},\cdots a_{n}\right]$ while $a_{c}=\left[{a_{1}}^{\prime },{a_{2}}^{\prime },\cdots {a_{n}}^{\prime }\right]$. The most direct way to measure the similarity between the two is to use the mean square error (MSE) loss based on the idea of logits matching as the loss function for $L_{transfer}$:

(2) $$L_{transfer}=\frac{1}{n}{\sum_{i=1}^{n}\left(a_{i}-{a_{i}}^{\prime }\right)}^{2}$$

During the training process of the target intelligent agents, the loss $L_{Q}$ calculated through environmental feedback will drive the agents to achieve the maximum expected reward in the environment during model updates, while the loss $L_{transfer}$ calculated through the teacher model will lead the agents to emulate the policy action outputs of the pre-trained intelligent agents in the target environment.

The training process is divided into two stages: the guiding stage and the specialization stage. In the guiding stage, the teacher policy model guides the current model to reuse knowledge. As training progresses, the agents gradually transition into the specialization stage, where the differences between tasks increase. Training depends on two factors: $L_{transfer}$ and $L_{Q}$. The transition between the two stages is achieved by gradually adjusting the hyperparameter α. The value of α ranges from [0, 1], and initially, it is initialized to a certain value, decreasing linearly as training progresses until it approaches 0. According to research related to reinforcement learning, retaining low-weight knowledge reuse can provide some noise for training to avoid task overfitting. Therefore, the hyperparameter α does not necessarily need to reach a value of 0 during training but decreases to a low weight.

### 3.3. Analysis of Transfer Framework Based on Improved Knowledge Distillation

#### 3.3.1. Network Structure

**Figure 5:**
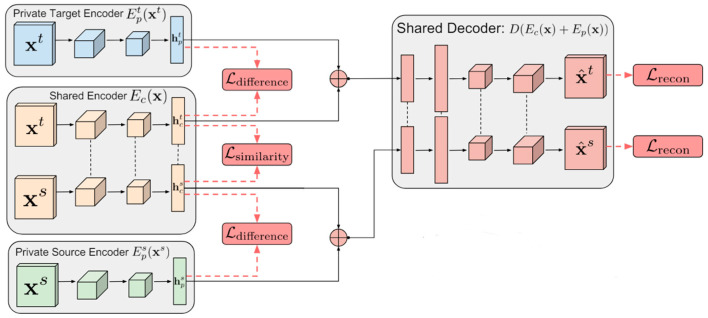
The architecture of domain separation network.

Based on the domain separation network (DSN), we hypothesize that in cross-domain transfer scenarios with an increasing number of intelligent agents, the differences in policy distributions between the source and target domains can be analyzed through the domain separation module. Because the differences in policy behavior of intelligent agents manifest in the mapping relationships between observation values and action values in different task domains, we aim to extract the maximum common features between action policy outputs of the source and target domains as much as possible through the DSN network. We consider this as supervised information that can guide the training of the target domain policy network, which is used to construct transfer loss to facilitate the training of the target policy network. The architecture of the DSN model is illustrated in Figure 5.

DSN comprises four modules: a common feature extractor $E_{c}$, source policy private feature extractor $E_{p}^{s}$, target policy private feature extractor $E_{p}^{t}$, and feature space reconstructor D. The common feature extractor utilizes a two-layer convolutional neural network and computes the similarity between the logits output by the source and target policy models using a similarity loss function. Its aim is to extract the common features between them as much as possible, resulting in the source policy common feature $h_{c}^{s}$ and the target policy common feature $h_{c}^{t}$. As this feature corresponds to the agents’ action execution, we consider that the extracted common features can demonstrate the commonalities between different strategies of the same agent across environmental changes. This common knowledge can be transferred among homogeneous agents because they exhibit interchangeability in the same task. In the knowledge distillation process described in the previous section, these common features will be used to replace the soft labels output by the teacher agent’s policy network.

The source policy private feature extractor $E_{p}^{s}$ and the target policy private feature extractor $E_{p}^{t}$ both employ two-layer convolutional neural networks to explicitly model the private features of the source and target policies’ outputs under the target environment observations, obtaining the source policy private feature $h_{p}^{s}$ and the target policy private feature $h_{p}^{t}$, respectively. The private parts of the policy output features under different tasks will be distinguished through difference loss.

The reconstructor (*D*) employs ReLU activation function and a four-layer convolutional neural network to merge and reconstruct the common and private features of agent policies under different tasks. By minimizing the reconstruction loss function, it maximizes the separation of common and private features between the source and target policies, ensuring the robustness of common feature extraction.

#### 3.3.2. Network Optimization Objectives

To effectively train each module of the DSN, four loss functions are jointly constructed to constrain the training process. Firstly, the difference loss function $L_{difference}$ is calculated based on the feature outputs of the source domain policy network and the target domain policy network, maximizing the differences in the common and private features between the two domains. Then, the similarity loss function $L_{similarity}$ is computed to maximize the similarity of the common features between the source and target domains, completing the feature extraction task. Subsequently, the extracted common and private features are inputted into the feature reconstructor, and the reconstruction loss function $L_{recon}$ is calculated to ensure the integrity and effectiveness of the features between the source and target domains. Finally, the common features from the source domain are used as shared knowledge to guide the training of the target network. Thus, the overall loss function for the DSN model of multi-agent knowledge transfer is as follows:

(3) $$L=L_{recon}+L_{similarity}+L_{difference}$$

The difference loss function $L_{difference}$ is utilized to compute the similarity between $h_{p}^{s}$ and $h_{c}^{s}$, $h_{p}^{t}$ and $h_{c}^{t}$, as well as between $h_{p}^{s}=h_{c}^{s}$ and $h_{p}^{t}=h_{c}^{t}$. $L_{difference}$ attains its maximum when $h_{p}^{s}$ and $h_{c}^{s}$ are orthogonal, as well as when $h_{p}^{t}$ and $h_{c}^{t}$ are orthogonal; conversely, it achieves its minimum. Thus, minimizing $L_{difference}$ enables the separation of $h_{p}^{s}$ and $h_{c}^{s}$, $h_{p}^{t}$ and $h_{c}^{t}$, facilitating the distinction between common and private features across the source and target domains. The computation formula is as follows:

(4) $$L_{difference}={\left\|h_{c}^{s^{T}}h_{p}^{s}\right\|}_{F}^{2}+{\left\|h_{c}^{t^{T}}h_{p}^{t}\right\|}_{F}^{2}$$

where ${\left\|\cdot \right\|}_{F}^{2}$ represents the square of the Frobenius norm. Fully separating $h_{p}^{s}$ and $h_{c}^{s}$, $h_{p}^{t}$ and $h_{c}^{t}$ through difference loss is not sufficient. The key to the DSN network module lies in ensuring that $h_{p}^{t}$ and $h_{c}^{t}$ are highly transferable, which requires adaptation between them. The calculation of the similarity loss function $L_{similarity}$ is as follows:

(5) $$\begin{array}{l} L_{similarity}=\frac{1}{{\left(N^{s}\right)}^{2}}\sum_{i,j=0}^{N^{s}}\kappa \left(h_{ci}^{s},h_{cj}^{s}\right)-\\ \frac{2}{N^{s}N^{t}}\sum_{i,j=0}^{N^{s},N^{t}}\kappa \left(h_{ci}^{s},h_{cj}^{t}\right)+\frac{1}{{\left(N^{t}\right)}^{2}}\sum_{i,j=0}^{N^{t}}\kappa \left(h_{ci}^{t},h_{cj}^{t}\right) \end{array}$$

where $\kappa \left(\cdot \;,\cdot \right)$ is the kernel function, which utilizes a linear combination of multiple radial basis function (RBF) kernels:

(6) $$\kappa \left(x_{i},y_{i}\right)=\sum_{n}\eta_{n}\exp\left\{-\frac{1}{2\sigma_{n}}{\left\|x_{i}-y_{i}\right\|}^{2}\right\}$$

where $\sigma_{n}$ is the standard deviation, and $\eta_{n}$ is the weight of the n RBF kernel.

The difference and similarity loss functions ensure the complete separation of $h_{p}^{s}$ and $h_{c}^{s}$, $h_{p}^{t}$ and $h_{c}^{t}$, while approximating the distribution of $h_{c}^{s}$ and $h_{c}^{t}$ to be equal. However, such separation operations compromise the integrity of the feature space. To ensure that the combination of common and private features constitutes a complete input, the following reconstruction loss function is constructed:

(7) $$L_{recon}=\sum_{i=1}^{N_{s}}L_{si\_mse}\left(x_{i}^{s},{\hat{x}}_{i}^{s}\right)+\sum_{i=1}^{N_{t}}L_{si_{mse}}\left(x_{i}^{t},{\hat{x}}_{i}^{t}\right)$$

This loss ensures that the common and private features between the source and target domains are distinct from each other without repetition. The calculation of $L_{si\_mse}$ is as follows:

(8) $$L_{si\_mse}\left(x,\hat{x}\right)=\frac{1}{\kappa }{\left\|x-\hat{x}\right\|}_{2}^{2}-\frac{1}{\kappa^{2}}\left(\left[x-\hat{x}\right]\cdot 1_{k}\right)$$

where x represents the input sample, $\hat{x}$ is the reconstructed sample, k is the dimensionality of the input sample vector, and $1_{k}$ is a column vector of length k with all values equal to 1.

## 4. Experimental Analysis

### 4.1. Experimental Environment Introduction

In this section, the construction of the simulation environment used to validate the multi-agent knowledge transfer algorithm is described, along with explanations of the objectives of each environment and the corresponding settings. The simulated environment is developed based on the multi-agent particle environment (MPE) for multi-agent particle environments. To extensively test the performance of the multi-agent knowledge transfer algorithm, this study conducted comparative experiments in both the multi-agent grid map environment developed by Jiang et al. and the complex interactive environment based on MPE [30]. For the complex interactive environment, this study combined the basic game environment of MPE with the multi-agent environment developed by Benjamin Black et al. and made certain modifications and designs on this basis [31]. The overall framework of the complex interactive environment is based on a basic physics engine, which considers the momentum of agents, ensuring that agents do not immediately move in the specified direction during interactions. Detailed descriptions of the environments for validating the knowledge transfer algorithm for homogeneous tasks in multi-agent collaborative systems are as follows:

#### 4.1.1. Unmanned Aerial Vehicle (UAV) Surveillance in Various Environments

**Figure 6:**
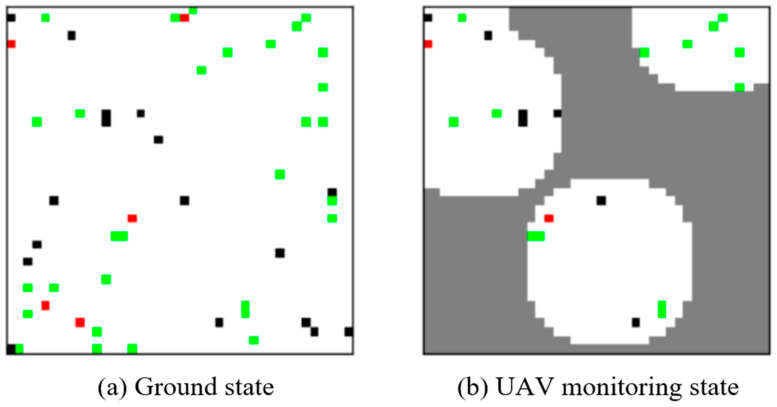
UAV surveillance environment.

This environment utilizes grid maps to simulate real-world scenarios where UAVs detect specific areas. It belongs to a multi-agent interactive environment with a discrete action space. Figure 6 illustrates the entire game environment, where the left image represents the ground scene, and the right image depicts the aerial reconnaissance view of UAVs. In the environment area, red squares represent pedestrians moving randomly in the white area, green squares represent trees, and black squares represent obstacles. Each UAV can freely fly in the area (overlapping positions are allowed) and has a locally defined field of view with a customizable radius. The collaborative goal of the UAV group is to monitor as many pedestrians as possible in the joint field of view, with higher rewards for detecting more pedestrians. Since the number of pedestrians exceeds the number of UAVs, the UAV group should minimize the overlap of their fields of view while exploring the entire area as much as possible. Additionally, UAVs cannot share observation results with each other, making this environment partially observable.

#### 4.1.2. Collaborative Target Point Occupation by Unmanned Aerial Vehicle (UAV)

**Figure 7:**
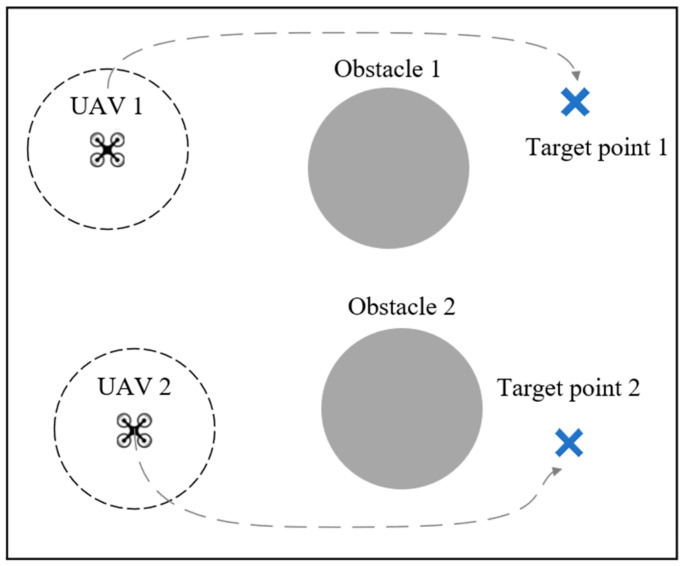
UAV cooperative target point occupation environment.

The UAV cooperative target point occupation environment is a partially observable environment where a certain number of UAVs in the scene must collaborate to approach target points without colliding, without communication, to minimize the distance between the targets and the corresponding UAVs, thereby maximizing the reward. If a UAV collides with obstacles, other UAVs, or scene boundaries, it incurs a penalty. Figure 7 illustrates an environment with two UAVs, two obstacles, and two target points. Each UAV has its own reconnaissance range and can obtain information about entities within its range. Target points are indicated by blue cross marks, while obstacles are represented by gray particles. Ideal flight paths for UAVs are depicted by gray dashed lines.

#### 4.1.3. UAV Cooperative Target Point Occupation Environment

**Figure 8:**
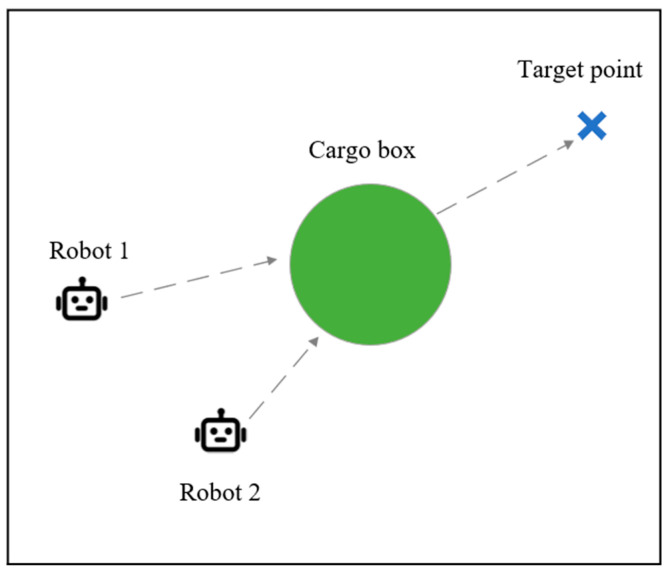
Cooperative robot pushing environment.

The cooperative robot pushing environment is a fully observable environment composed of multiple intelligent robots, a cargo box, and a marked target position. Figure 8 demonstrates a scenario where two robots cooperate in pushing a cargo box. Green particles represent the box to be pushed, while blue cross marks indicate the target position. Dashed lines represent the ideal collaborative strategy path for robots. The two robots need to push the box to the target position as quickly as possible. Each robot can observe the positions and velocities of all entities in the environment as well as the target. The box can only be pushed when all robots come into contact with it, and the direction of box movement aligns with the resultant force direction of the robots. The global reward obtained by robots is related to the distance between the box and the target point; the closer the distance, the higher the reward.

#### 4.1.4. UAV Cooperative Target Point Strike Environment

**Figure 9:**
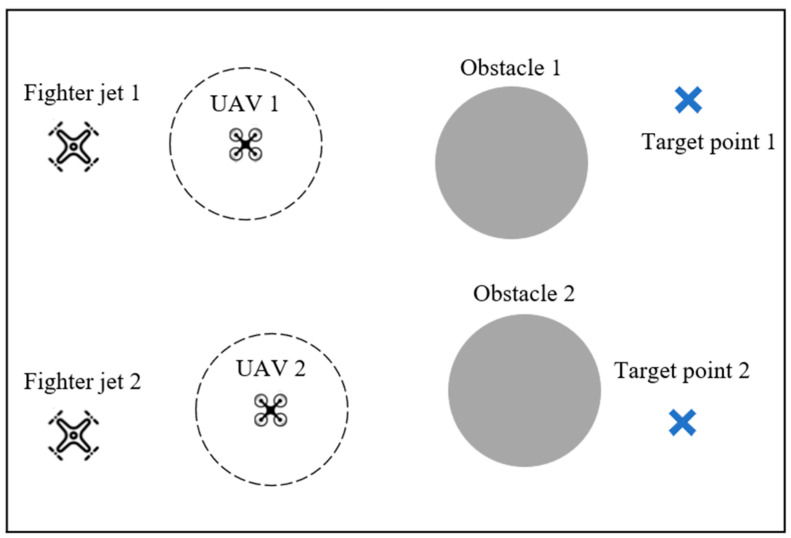
UAV cooperative target point strike environment.

The UAV cooperative target point strike scene is a partially observable environment where the UAV group is divided into two different types: combat units and reconnaissance units. Both types of units need to cooperate to approach designated target points for strikes. Reconnaissance aircraft observe information in the environment based on their reconnaissance range and send discrete communication messages to paired combat aircraft. Each combat aircraft depends on reconnaissance messages to conduct strikes on corresponding types of target points. In each round, combat aircraft listen to the communication signals from their paired reconnaissance aircraft as part of their state information, maintaining communication during interaction with the environment, but with limited communication, restricted to a few discrete action signals. The entire combat system maximizes global rewards by bringing combat aircraft closer to their designated target points. Collisions between any UAVs or collisions with obstacles incur penalties. The scene, as shown in Figure 9, uses dashed circles to represent the reconnaissance range of the reconnaissance aircraft, blue cross marks for corresponding strike target points, and gray particles for obstacles.

#### 4.1.5. Multi-Agent Cooperative Material Recovery Environment

**Figure 10:**
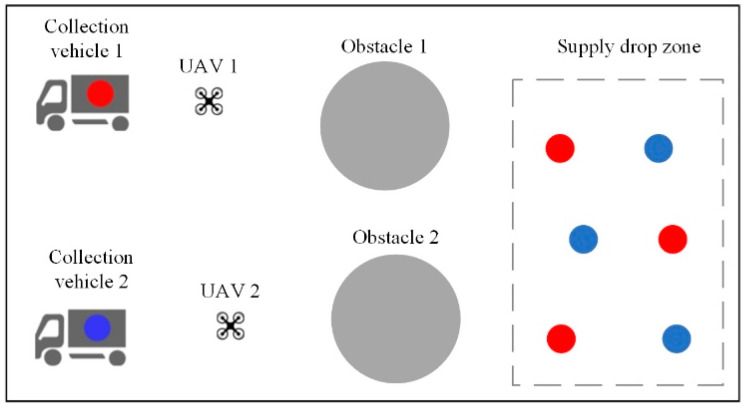
Multi-agent cooperative material recovery scene.

The multi-agent cooperative material recovery scene is a fully observable environment, consisting of two different types of agents: UAVs capable of carrying one piece of material at a time and collection vehicles for storing each type of material. Various types of materials need to be searched and collected by UAVs, with a limited number of each type available in the scene. When a piece of material is obtained by a UAV, a new piece of the corresponding type of material is randomly generated in the drop-off area of the map until all materials are collected. UAVs can transport any type of material but can only carry one piece at a time and must transfer it to the corresponding type of collection vehicle after obtaining it. Collection vehicles move slower than UAVs and cannot move to the material drop-off area. The overall objective of the task is for UAVs to collect as many materials as possible within a certain time frame and transport them to the corresponding collection vehicles, so both UAVs and collection vehicles need to plan their action trajectories reasonably based on real-time conditions. UAVs incur penalties for collisions, and penalties are also imposed on collection vehicles of different types if they are too close to each other. Each UAV receives a reward when it collects material, and when the material is successfully delivered to the corresponding collection vehicle for storage, the collection vehicle receives a reward. Figure 10 illustrates an environment with two UAVs, two collection vehicles, and two scene obstacles, with different types of materials represented by particles of different colors, corresponding to the collectors. The maximum number of each type of material present in the drop-off area is three.

### 4.2. Experimental Set

The partial parameter settings of the algorithms for the transfer experiments are shown in Table 1.

To evaluate the performance of the proposed knowledge transfer method using the domain separation network, various types of transfer tasks are designed for different experimental environments, as described in Table 2.

Task 1 involves transfer experiments in a UAV surveillance scenario. In the source task, there are 4 UAVs and 5 pedestrians, with a map size of 50. In the target task, there are 7 UAVs and 9 pedestrians, with a map size of 100. During the transfer process, the UAVs’ field of view remains consistent, with different joint state spaces but unchanged agent–task relations.

**Figure 11:**
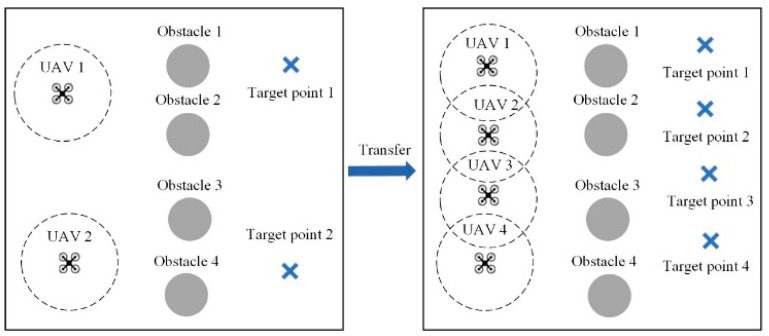
Illustration of transfer task 2.

Task 2 entails transfer tasks in a UAV cooperative target point occupation environment, as illustrated in Figure 11. In the source task, there are two UAVs, four obstacles, and two landmarks. The source task involves training two UAVs using the multi-agent deep deterministic policy gradient (MADDPG) algorithm, and the recorded policy models are used in the target task with four UAVs corresponding to the target points.

**Figure 12:**
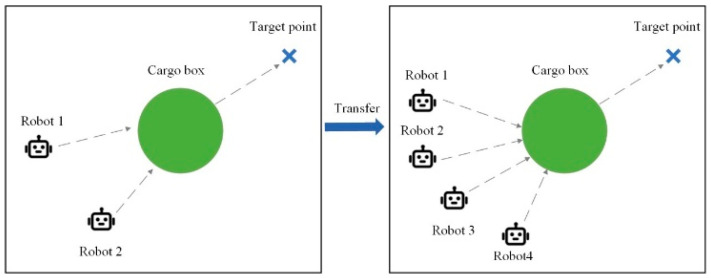
Illustration of transfer task 3.

Task 3 involves transfer tasks in a cooperative robot pushing environment, as depicted in Figure 12. The source task comprises two robots, while the target task scene involves four robots. The target task is more challenging, requiring all four robots to exert force simultaneously to move the box. This ensures that the task cannot be accomplished without cooperation. Additionally, the robots must coordinate the direction of force to move the box in a straight line, maximizing rewards. This presents the problem of delayed rewards, as positive rewards are only possible when all four robots take action on the box. Due to the sparsity of rewards, this transfer task effectively tests whether the algorithm can effectively guide agents to reduce blind exploration during the transfer process.

**Figure 13:**
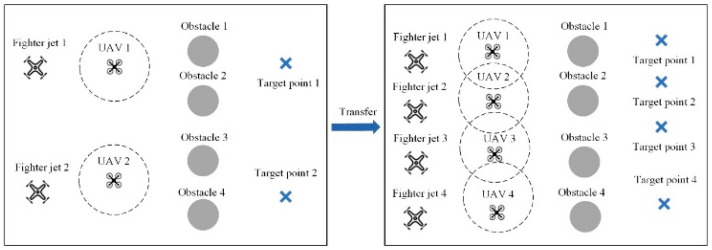
Illustration of transfer task 4.

Task 4 involves transfer tasks in a UAV cooperative target point strike environment, as shown in Figure 13. In the source task, there are two reconnaissance aircraft and two combat aircraft, with two combat aircraft needing to reach their respective strike points while avoiding four obstacles. In the target task, there are four reconnaissance aircraft and four combat aircraft, with the number of strike points corresponding to the number of combat aircraft.

**Figure 14:**
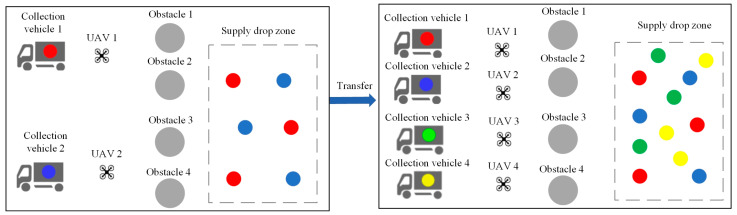
Illustration of transfer task 5.

Task 5 comprises transfer tasks in a multi-agent cooperative material recovery environment, as depicted in Figure 14. In the source task, there are two UAVs and two collection vehicles, with a maximum of three units for each type of material and a total of twenty units for each material type, along with four obstacles. In the target task, there are four UAVs and four collection vehicles, with the requirement to collect four different types of materials, maintaining the same limits and totals for each material type as in the source task.

In the aforementioned transfer experiments, the actor networks of the agents in the target tasks are initialized and updated according to different methods, while the critic networks are randomly initialized and retrained during interaction with the environment. In the transfer experiments, the policies of multiple agents in the source scene are trained using the MADDPG algorithm to achieve optimal performance. Subsequently, their policy networks are adjusted and matched with the agents in the target tasks, and knowledge transfer algorithms are employed to train the corresponding agents in the target environment. In the same transfer experiment, the initial positions of agents, obstacles, and target points are randomly initialized using different random seeds. To obtain relatively stable results, each transfer task is trained using 20 different random seeds, and the average reward curves from 20 training runs are evaluated to assess the effectiveness of model learning.

### 4.3. Analysis of Hyperparameter Impact on Transfer Performance

The previous subsection elucidated the trade-off between knowledge reuse and environmental exploration during agent training in the target task with hyperparameter α. Specifically, when α equals 1, it signifies complete utilization of the source policy’s knowledge in training the agents for the target task, while α equaling 0 indicates agents solely adjust their policy networks based on environmental feedback without reusing knowledge from the source policy.

**Figure 15:**
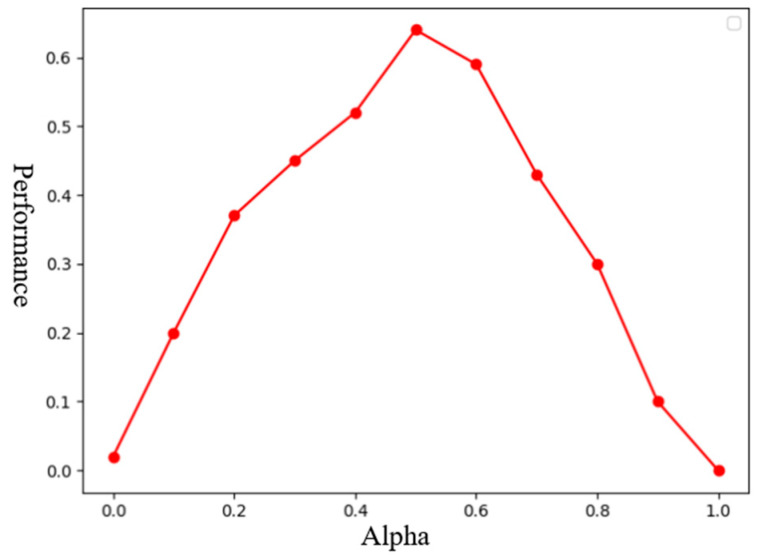
Illustration of transfer task 3. Performance comparison of the model under different values of α.

To investigate the influence of this parameter on the transfer effectiveness and select an appropriate value for α, we design experiments to assess the sensitivity of the model parameters. We intend to explore the initialization value of the impact of parameter α on overall performance as follows:

(9) $$T=\left\{T_{\alpha 1},T_{\alpha 1},T_{\alpha 1}\cdots T_{\alpha n}\right\}$$

(10) $${\mathrm{Performance}}_{\alpha }=\ln\left(\frac{T_{\max}}{T_{\alpha }}\right)$$

where $T_{\alpha }$ denotes the moment when the entire model achieves optimal performance using α training. *T* represents the set of $T_{\alpha }$, where $T_{\max}$ is the maximum value of set *T* and ${\mathrm{Performance}}_{\alpha }$ denotes the performance of α action scenario, with α parameter values incrementing from 0 to 1 by intervals of {0, 0.1, 0.2, 0.3, 0.4, 0.5, 0.6, 0.7, 0.8, 0.9, 1}. The results are illustrated in Figure 15.

Overall, when α is greater than 0.1, the transfer performance is significantly better than without transfer. Knowledge transfer processes exhibit notable improvements when α ranges from 0.4 to 0.6, with nearly maximum performance achieved at α equals 0.5. Consequently, for the subsequent experiments, we set the value of α to 0.5.

### 4.4. Comparative Experiments

To validate the effectiveness of the proposed method, this section sets up the following comparison groups:

1. MADDPG [32]: Not using transfer learning methods. This method directly retrains the agents in the target domain.
2. Finetune [33]: Training the target agents using the MADDPG reinforcement learning algorithm, initializing the agent network weights before training by adjusting the network structure to reuse the weights of the agent network trained in the source domain.
3. KD [29]: Direct knowledge distillation transfer method, guiding the learning of target agents by using the action output of the pre-trained policy as soft labels.
4. DSN-KD: The domain separation network proposed in this paper, explicitly modeling and separating the common features and private features of the source and target domains and using the extracted common features as common knowledge for transfer.

**Figure 16:**
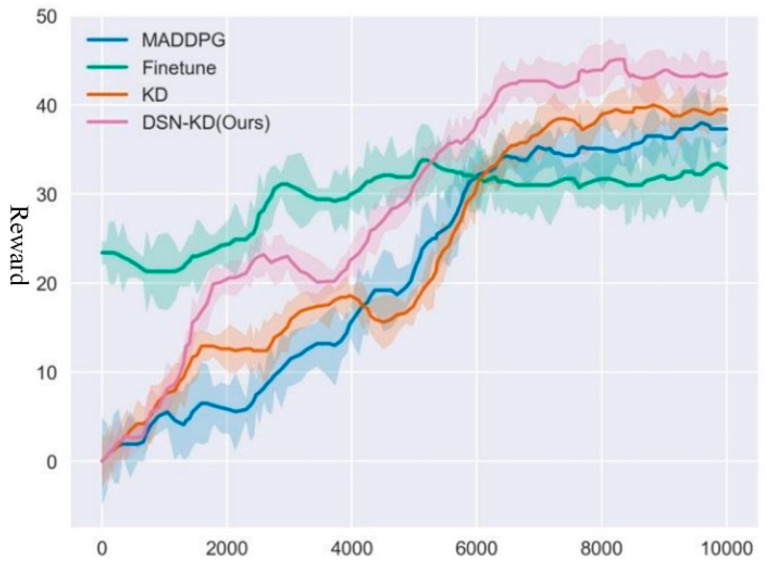
Comparative experiment for transfer task 1.

Firstly, experiments are conducted for Transfer Task 1. The effectiveness of training is measured by the average step rewards obtained by all agents from the environment in each round. The higher the reward, the better the model performance. The experiment is set with 10,000 rounds, each comprising 50 time steps, and the results are illustrated in Figure 16. The specific results of Experiment 1 are detailed in Table 3.

Table 3 reveals significant differences among various methods in terms of final reward, peak time, and threshold time. Although the MADDPG method retrains agents within the target domain, its training process is slower and results in lower final rewards due to the lack of transfer learning advantages. The Finetune method shows good initial jump-start performance, achieving a high initial reward (average of 23.4). However, as training progresses, the model tends to overfit, making it difficult to find the optimal strategy for the target task, resulting in a lower final reward. The KD method, by using knowledge distillation to transfer pre-training strategies, enables the target agent to effectively leverage the pre-trained model’s knowledge, achieving higher final rewards and demonstrating good stability during training. The proposed DSN-KD method, by employing a domain separation network, explicitly models and separates the features of the source and target domains, effectively utilizing common features for knowledge transfer. This method surpasses the KD method in overall learning speed and achieves the highest final reward, excelling in both threshold time and asymptotic performance. Experimental results indicate that the DSN-KD method outperforms other compared methods across multiple metrics, validating its effectiveness and advantages in transfer learning tasks. Compared to traditional methods like MADDPG and Finetune, DSN-KD not only accelerates training speed and improves final reward values but also effectively avoids overfitting issues. Although the KD method also shows good transfer performance, it is still inferior to DSN-KD in terms of final rewards and learning speed. Comprehensive analysis and comparison of experimental results demonstrate the powerful performance and broad application potential of the DSN-KD method in transfer learning tasks.

**Figure 17:**
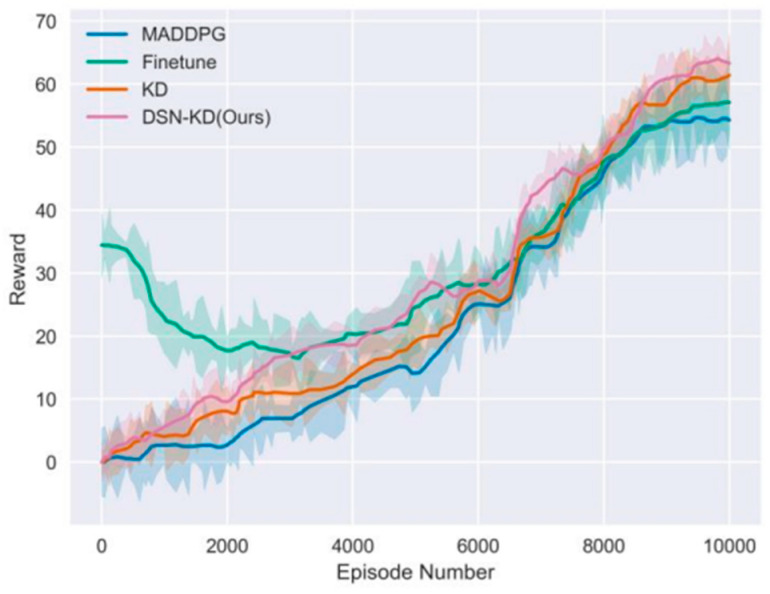
Comparative experiment for transfer task 2.

For Transfer Task 2, the experiment was conducted with 10,000 rounds to ensure model convergence. The average reward curve from multiple experiments is depicted in Figure 17.

In the comparative experiment of Task 2, the MADDPG algorithm achieved an average total reward convergence of around 60.2, requiring approximately 8900 rounds to reach satisfactory performance. Conversely, knowledge transfer methods based on fine-tuning converged in about 9800 rounds, yielding a final model reward of 57.8. The DSNKD transfer method converged to optimal performance in approximately 7800 rounds, with a final average reward of 67.5. Detailed data for the comparative experiment is provided in Table 4.

The experimental results indicate that DSN-KD initially possessed a faster and more robust learning speed compared to non-transfer scenarios. Moreover, as interaction continued, the DSN-KD training model required fewer rounds to converge to optimal performance, indicating successful knowledge reuse. Notably, when initializing agents with a model fine-tuned from the source task strategy, although performing well in the initial stages compared to MADDPG, no significant improvement in results was observed. This phenomenon is attributed to the model’s tendency to excel only in training scenarios and become ineffective when tasks change due to overfitting. Furthermore, the Finetune method exhibited considerable fluctuations during training, particularly in the early stages, resulting in noticeable performance declines. DSN-KD reached the convergence performance threshold set by the MADDPG algorithm in approximately 8200 steps, exhibiting superior performance and lower fluctuations compared to others. Direct knowledge distillation methods also achieved effective transfer in improving learning speed and optimal performance, owing to the minimal dissimilarity between source and target task knowledge. Additionally, the DSN-KD method exhibited superior initial learning speed compared to direct knowledge distillation and maintained stable exploration with excellent properties of the original strategy, thereby improving optimal performance. Based on the above analysis, the proposed DSN-KD method outperforms other compared methods across multiple metrics. This indicates the effectiveness and advantages of the DSN-KD method in transfer learning tasks. Compared to traditional methods like MADDPG and Finetune, DSN-KD not only accelerates training speed and improves final reward values but also effectively avoids overfitting issues. Although the KD method also shows good transfer performance, it is still inferior to DSN-KD in terms of final rewards and learning speed.

**Figure 18:**
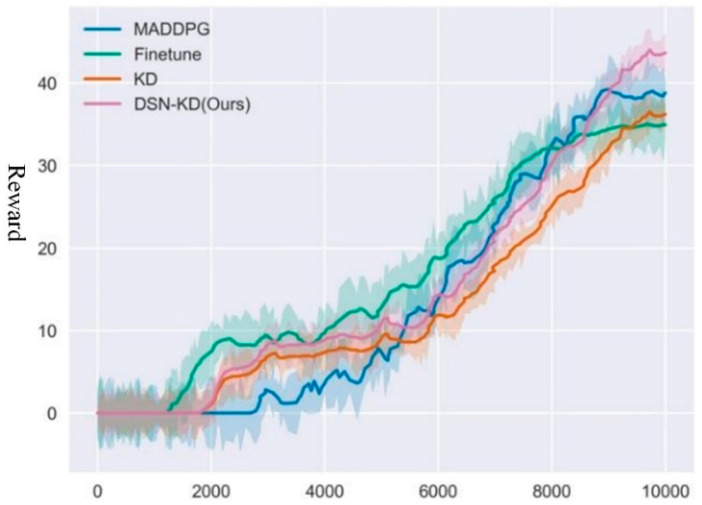
Comparative experiment for transfer task 3.

The comparison experiments for Transfer Task 3, likewise, measured training effectiveness based on average step rewards obtained in each round. A total of 10,000 rounds were conducted, with the reward curve depicted in Figure 18. Detailed data for the comparative experiment of transfer task 3 is presented in Table 5.

Compared to task 2 scenarios, task 3 conditions posed greater challenges, with the agent’s policy model more prone to converging to suboptimal strategies due to the highly unstable direction of combined force when pushing heavy boxes. Frequent performance declines during training indicate significant impacts from environmental instability, affecting both MADDPG retraining and fine-tuning based on the original strategy. However, under the guidance of an approximate optimal strategy from the source task, the DSN-KD method effectively achieved sustained learning and performance improvement. In terms of asymptotic performance metrics, the DSN-KD model’s final reward value of 43.3 indicates that knowledge transfer led to paths of multiple robots cooperating to push heavy boxes more closely resembling the ideal straight line. The optimal model’s final reward value obtained from the Finetune method was 34.7, demonstrating a performance gap compared to models trained directly from scratch. The KD method yielded a final model performance of 36.4, also exhibiting instances of negative transfer. Using the model convergence performance of the baseline reinforcement learning algorithm as a threshold, DSN-KD reached the threshold in approximately 8700 rounds. Although no significant improvement in learning speed was observed, DSN-KD demonstrated enhancement in initial performance compared to MADDPG and KD methods, maintaining effective exploration leveraging the superior properties of the original strategy throughout subsequent learning processes, thereby improving optimal performance.

**Figure 19:**
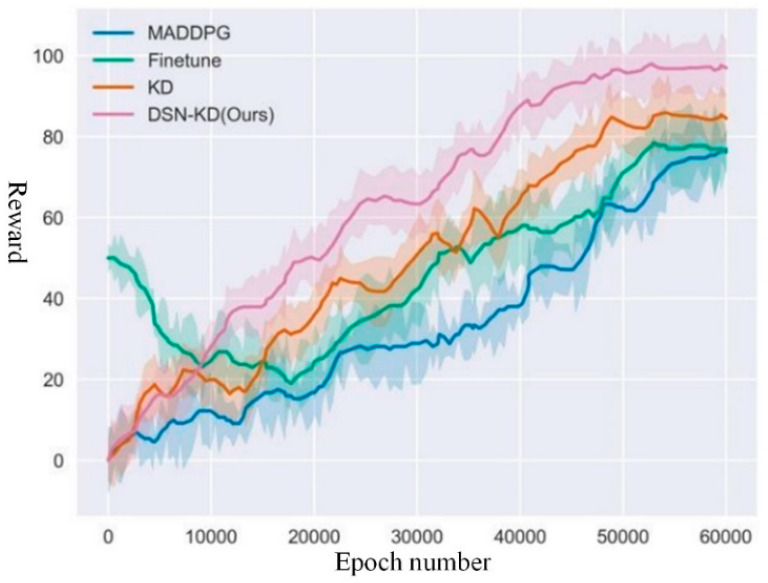
Comparative experiment for transfer task 4.

For transfer task 4, the total number of rounds was set at 60,000 to ensure optimal execution strategies in complex collaborative scenarios, with the reward curve illustrated in Figure 19. Detailed data for the comparative experiment of transfer task 4 is provided in Table 6.

The experimental results indicate that the policy model’s effectiveness obtained through MADDPG retraining and fine-tuning based on the original strategy were highly comparable. Meanwhile, DSN-KD and KD methods achieved positive transfer. Under the guidance of the source task strategy and agent collaboration, effective performance improvements were realized. In terms of asymptotic performance, the DSN-KD model achieved a final performance of 96.3, significantly outperforming other methods. This suggests that the joint strategies learned by the DSN-KD method within a limited number of time steps bring combat units in the task closer to the target point. Using the model’s final performance obtained from the baseline reinforcement learning algorithm as a threshold, the threshold time for DSN-KD was approximately 34,000 rounds, representing a significant improvement compared to other methods.

**Figure 20:**
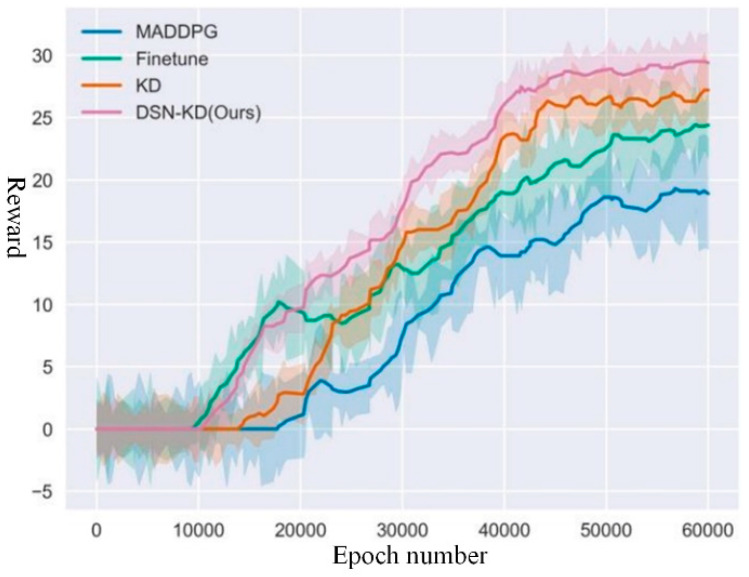
Comparative experiment for transfer task 5.

For Transfer Task 5, with a total of 60,000 rounds, the reward curve is depicted in Figure 20. Detailed data for the comparative experiment of Transfer Task 5 is presented in Table 7.

In the experimental setting of Task 5, the direct training effect of MADDPG was poor, attributed to environmental factors where reinforcement learning training under sparse reward conditions is challenging to guarantee. All transfer methods achieved positive transfer effects, indicating improvements in the agent’s blind exploration to a certain extent. In terms of asymptotic performance, the DSN-KD model achieved a final performance of 29.3, representing effective improvements compared to the Finetune method’s model performance of 24.4 and the KD method’s performance of 26.9. In other words, the action strategies of collection vehicles and drones led to the collection of more resources within a limited time. Using the model’s final performance obtained from the baseline reinforcement learning algorithm as a threshold, the threshold time for DSN-KD was approximately 31,500 rounds, compared to 39,000 for the Finetune method and 38,000 for the KD method.

Based on the results from the five experimental groups, the proposed knowledge distillation framework, DSN-KD, demonstrates superior performance compared to the other three methods. The primary reasons include: (1) By explicitly modeling and separating common and private features of the source and target domains, DSN-KD more effectively leverages useful information from the source domain for knowledge transfer. Common features are extracted and transferred as shared knowledge, enabling the target domain to quickly adapt to new tasks and achieve higher reward values early in the training process. In contrast, the MADDPG method retrains agents directly in the target domain without utilizing knowledge from the source domain, resulting in longer training times and lower performance. The Finetune method, although attempting to reuse network weights from the source domain, suffers from overfitting, leading to instability in later stages of training. The KD method, while employing knowledge distillation for transfer, fails to explicitly separate features, resulting in limited performance improvement. (2) In transfer tasks, the DSN-KD method exhibits faster learning speeds and higher final reward values. By separating common and private features, DSN-KD reduces interference from irrelevant features on the target task, thereby enhancing learning efficiency. Additionally, DSN-KD shows smaller fluctuations during training, indicating strong stability in handling different tasks. The MADDPG method, due to starting from scratch in training agents, has slower learning speeds and lower final reward values. The Finetune method performs well initially but is prone to overfitting and performance fluctuations as training progresses. The KD method, although improving learning speed, still requires a longer time to converge after reaching optimal performance, with certain instability during training. (3) By utilizing common features between the source and target domains, the DSN-KD method not only improves initial learning speed but also excels in final reward value and convergence speed. Experimental results indicate that the DSN-KD method achieves optimal performance in transfer tasks, and after reaching the highest reward, it does not experience performance degradation, demonstrating robust stability. The MADDPG method shows poorer convergence performance, requiring more time to achieve better results. The Finetune method shows limited performance improvement in later training stages and is prone to overfitting. The KD method shows improvements in learning speed and final reward value but does not surpass DSN-KD in all aspects.

## 5. Conclusions

This paper addresses the issue of the inability to directly reuse source policies in homogeneous task transfer by proposing a multi-agent knowledge transfer algorithm based on enhanced knowledge distillation. In this method, a domain-separated network is utilized within a multi-agent collaborative environment to extract common knowledge from the policies of pre-trained agents in the source task, which effectively guides the training of agents in the target task. Subsequently, the algorithm’s performance was validated using scenarios constructed on the MPE platform to assess its effectiveness across various settings, including scenarios with varying numbers of agents. The experimental results from both existing and designed scenarios demonstrate the significant enhancement in training speed and optimal policy performance achieved by the proposed DSN-KD transfer algorithm.

Future work will explore how the algorithm performs in different domains, such as autonomous driving, robotic swarm systems, and real-time strategy games, to establish its versatility. Developing adaptive knowledge distillation techniques that can dynamically adjust the knowledge transfer process based on the target task’s complexity and similarity to the source task could further improve transfer efficiency and effectiveness.

## Figures and Tables

**Table 1:** Partial parameter settings of DSN-KD.

Parameter Name	Parameter Value	Parameter Description
γ	0.99	Discount factor
batch-size	1024	Batch size
buffer-size	1,000,000	Experience replay buffer capacity
learning_rate	0.0005	Agent network learning rate
hidden_dim	128	Number of hidden layer neurons
num_updates	4	Parameter update interval across round
steps_per_episode	50	Maximum number of steps per episode

**Table 2:** Settings of homogeneous transfer tasks.

Index	Scene	Source Task	Target Task
Task 1	Drone surveillance	4 drones, 5 pedestrians	7 drones, 9 pedestrians
Task 2	Drone cooperative target occupation	2 drones, 2 target points	4 drones, 4 target points
Task 3	Collaborative robot box pushing	2 robots	4 robots
Task 4	Drone cooperative target strike	2 fighters, 2 scouts	4 fighters, 4 scouts
Task 5	Multi-agent cooperative resource retrieval	2 drones, 2 collection vehicles	4 drones, 4 collection vehicles

**Table 3:** Experimental results for task 1 comparison.

Method Name	Final Reward	Peak Time	Threshold Time
MADDPG	36.2	9800	10,000
Finetune	32.5	5200	-
KD	40.0	9400	5900
DSN-KD(Ours)	42.6	7800	6900

**Table 4:** Experimental results for task 2 comparison.

Method Name	Final Reward	Peak Time	Threshold Time
MADDPG	54.3	8500	10,000
Finetune	57.1	10,000	8900
KD	61.4	10,000	8100
DSN-KD(Ours)	63.5	9800	8200

**Table 5:** Experimental results for task 3 comparison.

Method Name	Final Reward	Peak Time	Threshold Time
MADDPG	38.9	8700	10,000
Finetune	34.7	9400	-
KD	36.4	9600	-
DSN-KD(Ours)	43.3	9600	8700

**Table 6:** Experimental results for task 4 comparison.

Method Name	Final Reward	Peak Time	Threshold Time
MADDPG	76.1	60,000	60,000
Finetune	76.2	52,000	51,000
KD	84.4	52,500	45,000
DSN-KD(Ours)	96.3	52,000	34,000

**Table 7:** Comparative experiment for transfer task 5.

Method Name	Final Reward	Peak Time	Threshold Time
MADDPG	18.2	56,000	60,000
Finetune	24.4	59,000	39,000
KD	26.9	60,000	38,000
DSN-KD(Ours)	29.3	57,000	31,500

## Data Availability

The authors have no permission to share the data.
